# Host–guest interactions between *p*-sulfonatocalix[4]arene and *p*-sulfonatothiacalix[4]arene and group IA, IIA and f-block metal cations: a DFT/SMD study

**DOI:** 10.3762/bjoc.15.131

**Published:** 2019-06-17

**Authors:** Valya K Nikolova, Cristina V Kirkova, Silvia E Angelova, Todor M Dudev

**Affiliations:** 1 Faculty of Chemistry and Pharmacy, Sofia University “St. Kl. Ohridski”, 1164 Sofia, Bulgaria; 2 Institute of Organic Chemistry with Centre of Phytochemistry, Bulgarian Academy of Sciences, 1113 Sofia, Bulgaria

**Keywords:** complex formation, DFT, group IA, IIA and f-block metal cations, macrocycles, *p*-sulfonatocalix[4]arene, *p*-sulfonatothiacalix[4]arene

## Abstract

The molecular recognition in aqueous solution is extremely important because most biological processes occur in aqueous solution. Water-soluble members of the calix[*n*]arene family (e.g., *p*-sulfonato substituted) can serve as model systems for studying the nature and manner of interactions between biological receptors and small ions. The complex formation behavior of water-soluble *p*-sulfonatocalix[4]arene and thiacalix[4]arene and group IA, IIA and f-block metal cations has been investigated computationally by means of density functional theory computations in the gas phase and in aqueous environment. The calculated Gibbs free energy values of the complex formation reaction of these ligands with the bare metal cations suggest a spontaneous and energy-favorable process for all metal cations in the gas phase and only for Na^+^, Mg^2+^, Lu^3+^ cations in water environment. For one of the studied cations (La^3+^) a supramolecular approach with explicit solvent treatment has been applied in the study of the effect of metal hydration on the complexation process. The La^3+^ binding to the *p*-sulfonatocalix[4]arene host molecule (now in the metal’s second coordination shell) is still exergonic as evidenced by the negative Gibbs free energy values (Δ*G*^1^ and Δ*G*^78^). The combination of implicit/explicit solvent treatment seems useful in the modeling of the *p*-sulfonatocalix[4]arene (and thiacalix[4]arene) complexes with metal cations and in the prediction of the thermodynamic parameters of the complex formation reactions.

## Introduction

If macrocycles are pillars of the supramolecular chemistry, then calixarenes (“calix” = vase + “arene”) are the 3rd pillar after the well-studied cyclodextrins and crown ethers. Calixarenes are products of phenol–aldehyde condensation, as the aromatic components may derived from phenol, resorcinol, or pyrogallol; the aldehyde most often used for phenol is simple formaldehyde (methanal, HCHO), while larger aldehydes (e.g., acetaldehyde – ethanal, CH_3_CHO) are required in condensation reactions with resorcinol and pyrogallol [1]. Thiacalixarenes are macrocycles (or cyclic oligomers) based on a condensation of the same phenol derivatives and sulfur [2]. They are characterized by a larger cavity size than the conventional calixarenes (with the same repeating units). The sulfur functionalities are stated to provide better metal complexation [3]. Unmodified calixarenes and thiacalixarenes are sparingly soluble, have chemical and thermal stability and act as host molecules as they possess cavities, but their inclusion properties are not as good as those of other common macrocycles. Two positions (phenolic OH groups and *p-*positions) in the calixarenes’ structure can be easily modified by subsequent reactions [4]. As a result, calixarenes have a great potential as simple scaffolds to build molecular receptors and multivalent ligands with novel features.

They have found various applications in chemical [5], analytical [6–9], and engineering materials fields (self-assembling monolayers, surfactants, sensors [10–11]), in polymer synthesis [12–13], controlled drug-delivery systems [14], and so on, besides the biochemical, biopharmaceutical, biological, biomimetic (enzyme mimics, transport across membranes, ion channels, etc.) and biomedical (in cancer chemotherapy) applications, reviewed by Atwood et al. [15], Perret et al. [16], Da Silva et al. [17], Nimse et al. [18], Guo et al. [19], Agrawal et al. [20], Yousaf et al. [21]. The biological activity of the calix[*n*]arenes on various life forms from viruses to human beings have been reported [22].

The first water-soluble calix[*n*]arene derivative, *p*-*tert*-butylcalix[4]arenetetracarboxylic acid, was synthesized in 1984 [23]. The first paper on the calixarenes having sulfonate groups (and demonstrating high aqueous solubility) was published in the same year by Shinkai et al. [24]. The first structural study of the sodium salt of *p*-sulfonatocalix[4]arene, was reported in 1988 by Atwood et al. [25]. The truncated square pyramid-shaped *p*-sulfonatocalix[4]arene possesses hydrophilic rims, separated by a hydrophobic mid-region, and adopts different conformations depending on different factors: the solvent, the nature of the guest molecule, the functionalization of the lower (OH) rim [16]. The 3D cavities and the π-electron-rich sulfonate groups endow them with fascinating affinities and selectivities; they have demonstrated excellent complex ability towards inorganic cations, organic ammonium cations, pyridinium guests, neutral molecules (alcohols, ketones, nitriles), dye molecules, etc. [26]. *p*-Sulfonatocalix[*n*]arenes are complexing agents for structurally diverse biologically active molecules [27], including some amino acids [28] and proteins [29]. They are also biocompatible: compared to other types of macrocyclic molecules such as cyclodextrins and cucurbiturils (which are also water soluble), *p*-sulfonatocalix[*n*]arenes do not exhibit any toxicity, which makes them applicable in medicine.

The binding affinities and thermodynamics of *p*-sulfonato-calix[4]arene upon complexation with different inorganic and organic cations in water have been investigated experimentally by Bonal et al. [30] and Morel et al. [31]. The experiments indicated 1:1 stoichiometry of the inclusion complexes and much weaker binding abilities for monovalent cations than for divalent and trivalent cations. In the study of the binding behaviors of some *p-*sulfonatocalix[4]arenes with inorganic monoatomic cations and organic ammonium cations by microcalorimetry the sulfonate groups of hosts were identified as anchoring points for the positively charged guests. Cation–π interactions between the monoatomic cations and *p*-sulfonatocalix[4]arene in water are supposed (but not proven) to take part in the inclusion complex formation [31].

Mendes et al. have carried out molecular dynamics (MD) simulations of association of *p*-sulfonatocalix[4]arene and some inorganic and organic cations in aqueous solution [32]. The predicted Δ*G* value (relative Gibbs energy) of the complexation between the host calixarene molecule and hydrated La^3+^ cation (with an average coordination number of water molecules in the first hydration shell of about 10), which is located outside the host cavity, has been found to be in agreement with the experimental data [32].

We report herein our computational (DFT) results on the complexation of *p*-sulfonatocalix[4]arene and thiacalix[4]arene with some metal guest cations. The thermodynamic descriptors of the group IA, IIA and f-block metal cations binding to the host calixarene systems have been evaluated and the factors that affect the interactions in the gas phase and in water medium have been unraveled.

**Scheme 1:**
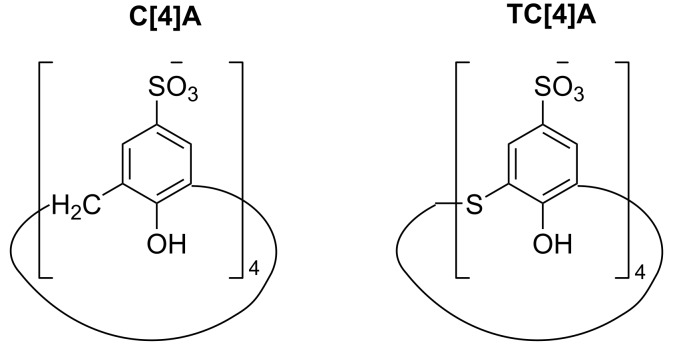
Schematic representation of the structures of *p*-sulfonatocalix[4]arene (**C[4]A**) and *p*-sulfonatothiacalix[4]arene (**TC[4]A**).

Available data on the experimental p*K*_a_ values [31,33–34] imply that in acidic water solution of pH ≈ 2 the host calixarene systems have all the sulfonic acid groups deprotonated and all the phenolic hydroxy groups protonated thus the anionic structures shown on Scheme 1 were modeled and employed in our computational studies.

Studies on the thermodynamic behavior and recognition processes of water-soluble calixarenes and cationic guests are important in understanding the possible cooperative/competitive contributions of different intramolecular interactions working between the host and guest species. Knowledge (at molecular level) of structural/functional information, energetics/thermodynamics of the binding event, complementarity of molecular shapes, etc., is useful for designing receptor molecules.

## Results and Discussion

**Figure 1:**
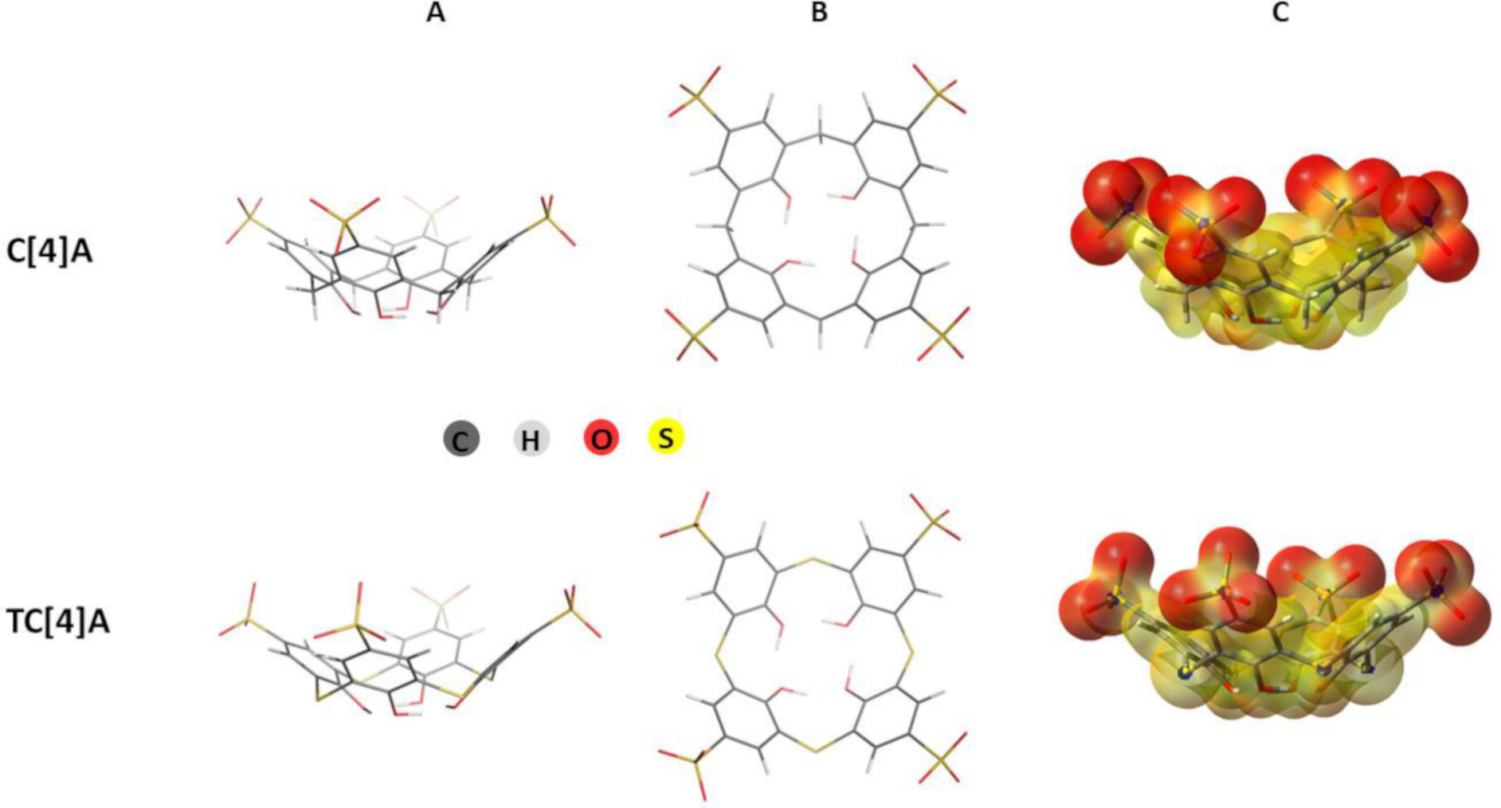
Optimized structures of negatively charged **C[4]A** and **TC[4]A**, presented in two projections: (A) side view and (B) view from the rim, trimmed with sulfonate groups (SO_3_ rim); (C) electron density of **C[4]A** and **TC[4]A** (isovalue = 0.002), mapped with electrostatic potential (color scheme: red/yellow for negative surface map values and blue for the positive ones).

M062X/6-31G(d,p) optimized structures of the host systems in cone conformation are presented in Figure 1 in two projections: side view and view from the rim trimmed with sulfonate groups. The optimized **C[4]A** and **TC[4]A** systems possess four-fold symmetry (Figure 1B). The four hydroxy groups surrounding the narrow rim (OH rim) are linked via hydrogen bonds in a tail–head arrangement.

### **C[4]A** and **TC[4]A** as first-shell ligands for IA/IIA/f-block metal cations

The formation of **[C[4]A–M]^(4−^*^n^*^)−^** and **[TC[4]A–M]^(4−^*^n^*^)−^** complexes (M = IA/IIA/f-block metal, *n* = 1–3) where calixarenes act as first-shell ligands to the metal cations, was studied.

**Figure 2:**
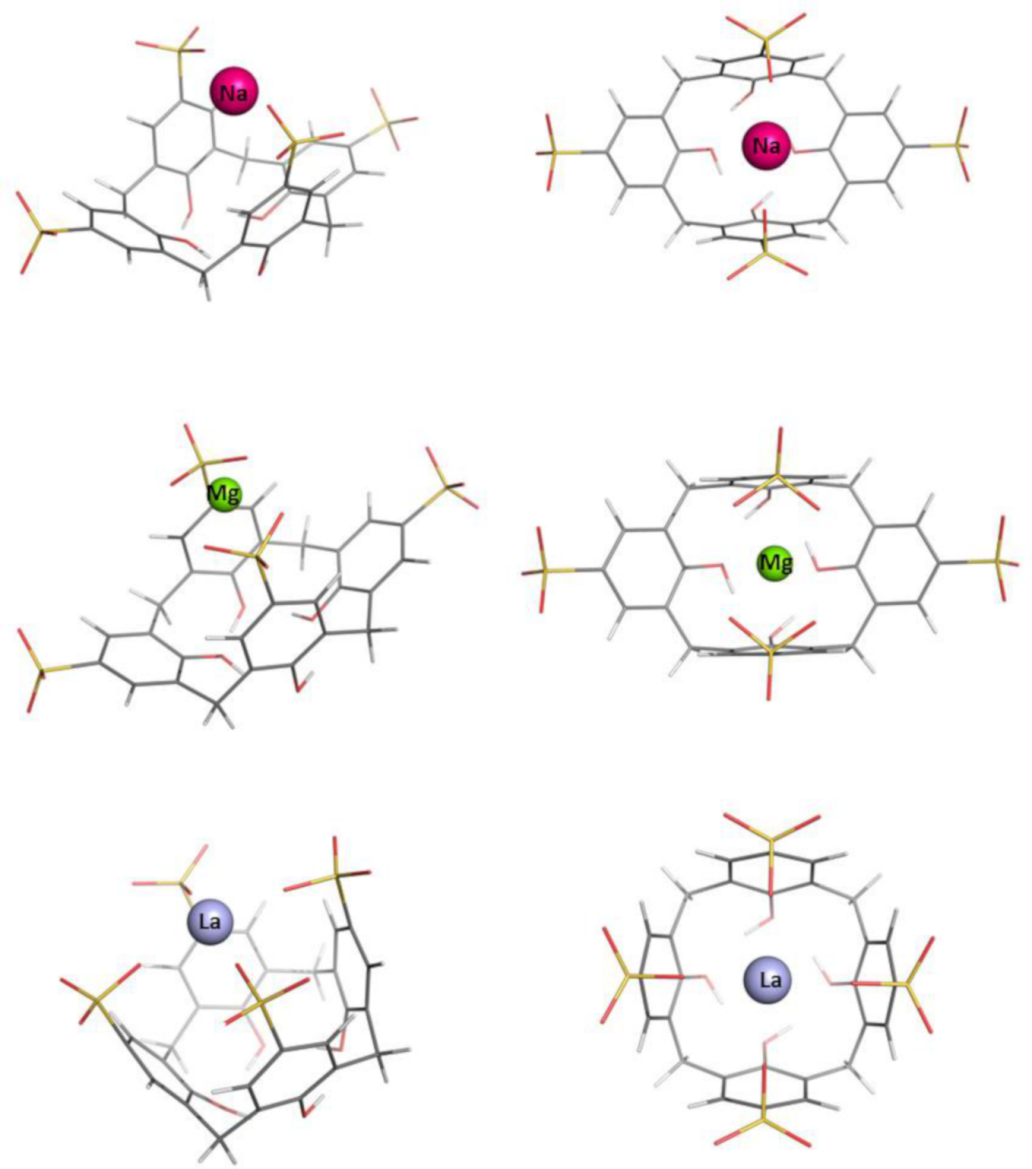
Optimized structures of **C[4]A** complexes with Na^+^, Mg^2+^ and La^3+^.

**Figure 3:**
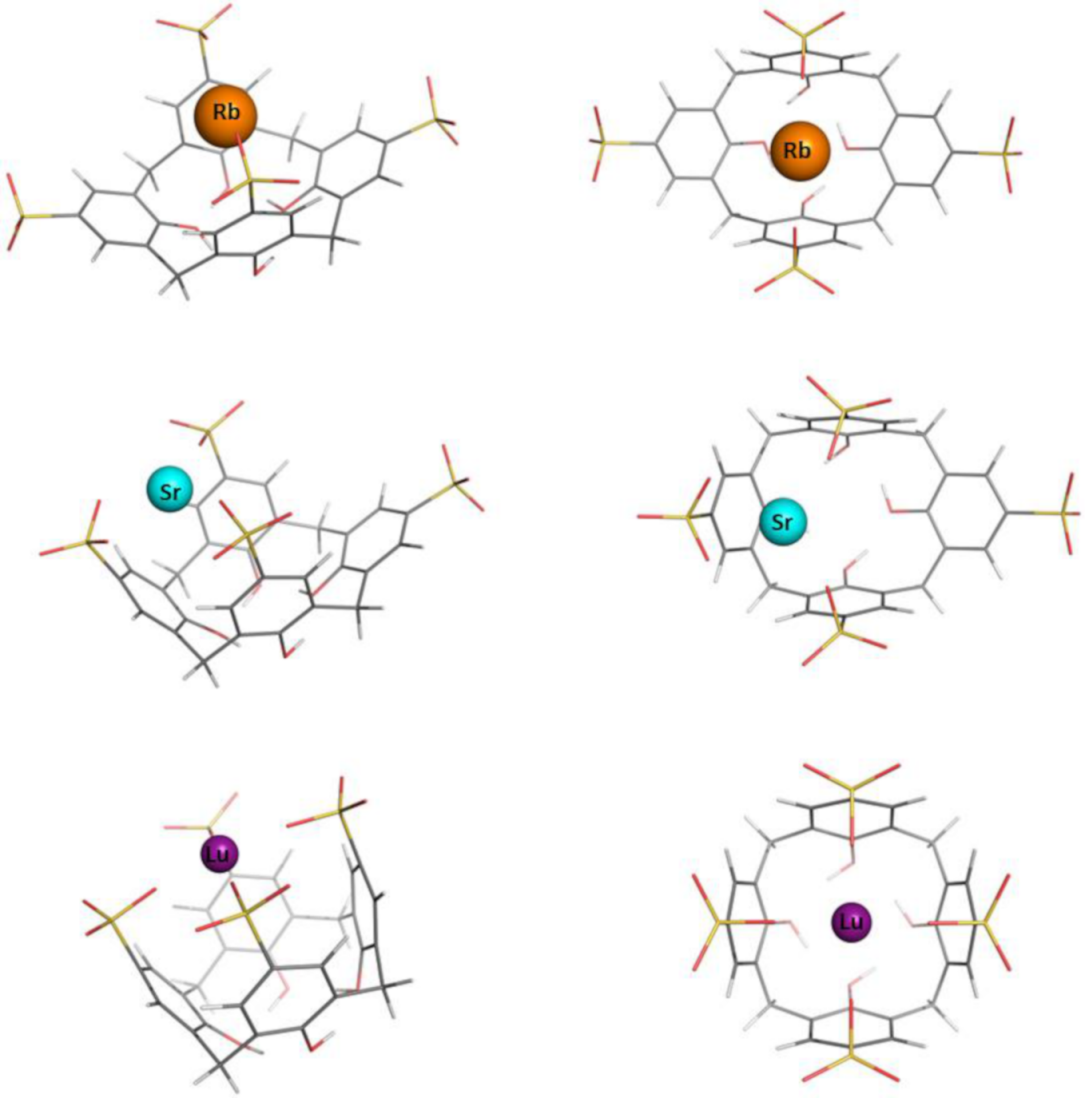
Optimized structures of **C[4]A** complexes with Rb^+^, Sr^2+^ and Lu^3+^.

The optimization of the **[C[4]A–Na]^3-^** structure was initiated from the geometry with the Na^+^ cation positioning inside the cavity, “above” the center of the OH trimmed rim plane (≈1 Å) of the optimized structure of the free **C[4]A**. In the optimization process the cation moves along the *z*-axis from its initial position toward the sulfonate groups. Upon reaching the level of the sulfonate groups the metal attracts two of them, which are oppositely placed. The other two sulfonate groups became more distant in the optimized structure of the complex (Figure 2). For the rest of the complexes, the starting conformation is built by locating the naked metal cation close to the sulfonate groups by using the optimized **[C[4]A–Na]^3−^** structure and replacing the metal. The optimized structures of the resultant **C[4]A**-based metal complexes with group IA, IIA and f-block metal cations are shown in Figure 2 and Figure 3 in two projections. The initial shape of the “empty” calixarenes (truncated square pyramid or popcorn box frustum) becomes distorted for all metal cations hosted. The coordination number of Na^+^ and Rb^+^ cations in the complex is equal to 2; that of Mg^2+^, La^3+^, Sr^2+^ and Lu^3+^ is 4. In the **[C[4]A−Na]^3−^** and **[C[4]A−Rb]^3−^** complexes the metal is bound to one oxygen atom from 2 opposite SO_3_^−^ groups. The Mg^2+^ ion also coordinates to 2 opposite SO_3_^−^ groups (to two oxygen atoms from each one). La^3+^ and Lu^3+^ ions, which are characterized with the same coordination number of 4 tend to coordinate to all 4 SO_3_^−^ groups. In the optimized **[C[4]A–Sr]^2−^** structure 3 of the SO_3_^−^ groups are involved in coordinative bond formation, as a result Sr^2+^ is tilted to one of the **C[4]A** walls (Figure 3).

**Figure 4:**
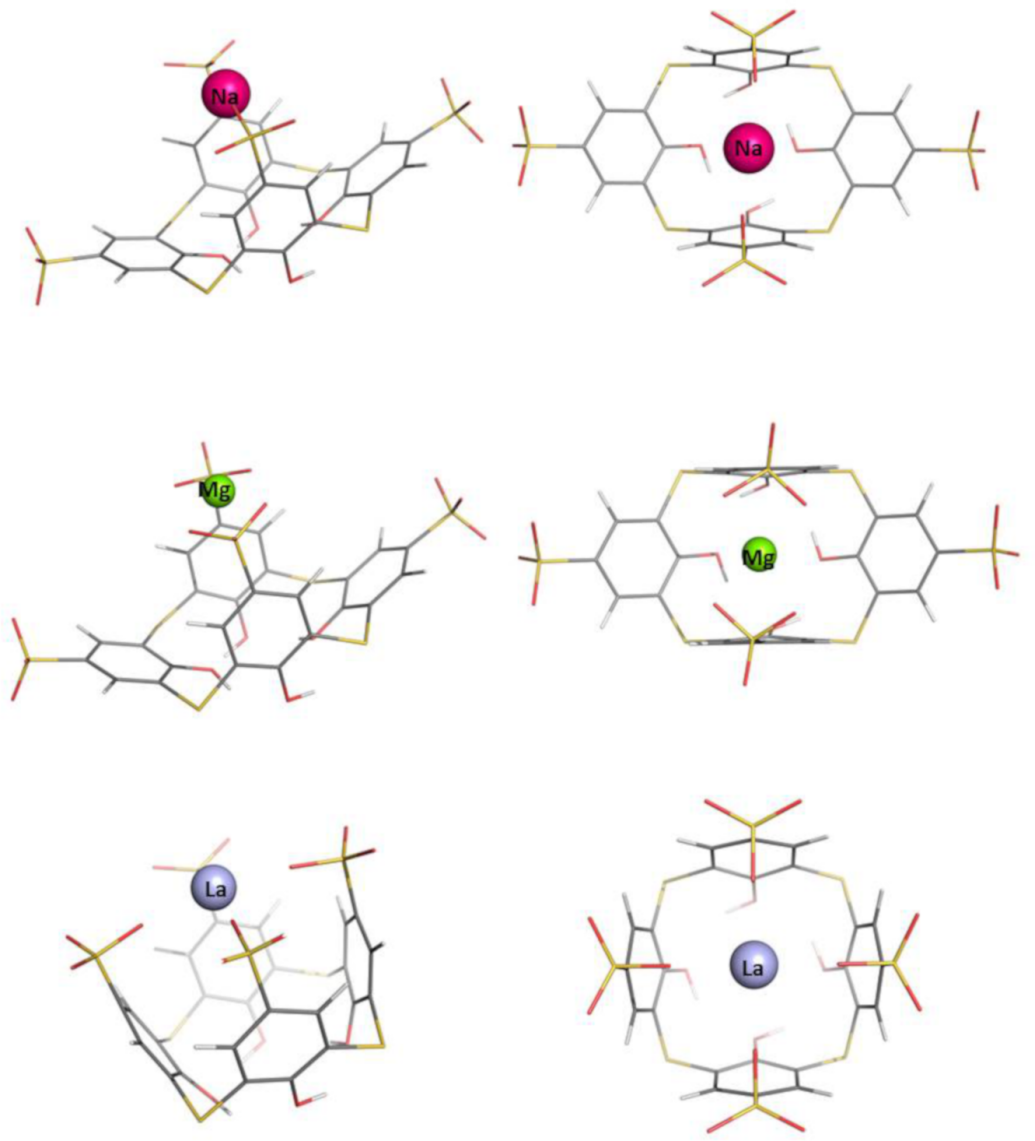
Optimized structures of **TC[4]A** complexes with Na^+^, Mg^2+^ and La^3+^.

**Figure 5:**
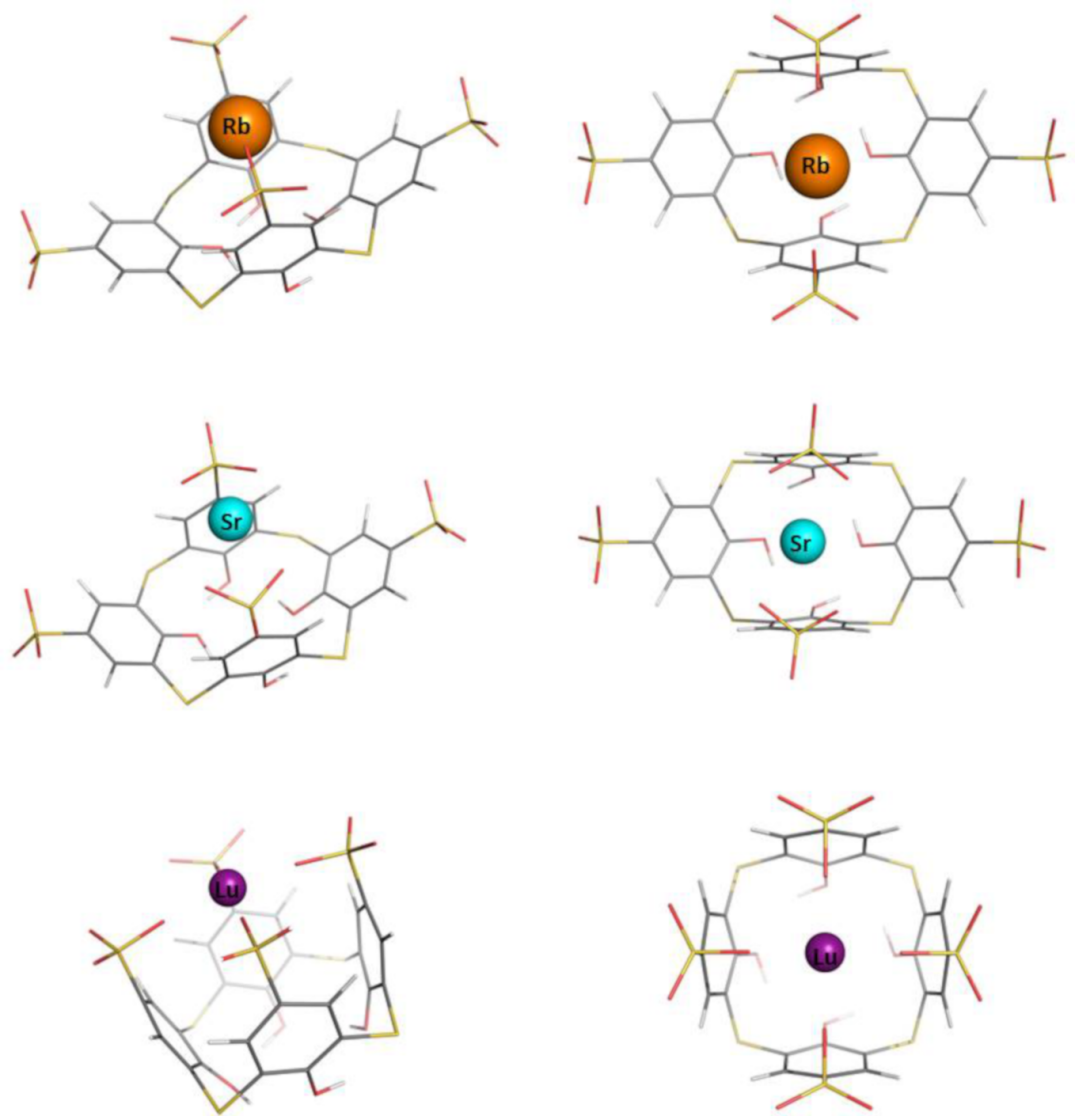
Optimized structures of **TC[4]A** complexes with Rb^+^, Sr^2+^ and Lu^3+^.

The optimization of Na^+^, Mg^2+^ and La^3+^-complexes with **TC[4]A** was initiated from the respective optimized **[C[4]A−M]^(4−^*^n^*^)−^** geometry where the CH_2_ groups were replaced by sulfur atoms. The Rb^+^, Sr^2+^ and Lu^3+^ complexes with **TC[4]A** were modeled from the optimized geometries of the Na^+^, Mg^2+^ and La^3+^ complexes. In the resultant **TC[4]A**-based complexes the metal cations are located as in the respective **C[4]A**-based complexes and have the same coordination numbers, except for the Sr^2+^ cation, which is moved back to the z-axis and is equidistant from the opposite **TC[4]A** walls (Figure 4 and Figure 5).

The Gibbs free energies of complex formation in the gas-phase (Δ*G*^1^) and water environment (Δ*G*^78^) are presented in Table 1. The quite large negative Δ*G*^1^ values indicate that all the complex formation reactions in the gas phase are exergonic (favourable). A significant effect of the metal’s charge on the energetics of the complex formation is observed. A rough correlation between the metal cation charges and ∆*G*^1^ values is observed: the predicted ∆*G*^1^ values increase significantly with increasing the oxidation state of the metal cation. There is no clear correlation between the cation radius (Table 2) and the energetics of the complex formation in the gas phase. Despite our initial expectations, although the **C[4]A** and **TC[4]A** calixarenes differ in composition, their ligating properties appear to be almost identical. This is because the binding centers are located in the SO_3_ belt (common structural unit for both molecules) which is far away from the structurally different methylene/sulfur bridges at the lower rim. Water solvation has great impact on the Gibbs energies of the complex formation. The process of inclusion complex formation in aqueous solution becomes less favorable with some Δ*G*s (for La^3+^, Rb^+^ and Sr^2+^) being shifted to a positive ground (Table 1).

**Table 1:** BSSE-corrected Gibbs free energies (in kcal mol^−1^) in the gas phase (Δ*G*^1^) and in water (Δ*G*^78^) for the **[C[4]A–M]^(4−^*^n^*^)−^** and **[TC[4]A–M]^(4−n)−^** (*n* = 1–3) complex formation reactions, **C[4]A** + M*^n^*^+^ → **[C[4]A–M]^(4−n)−^** and **TC[4]A** + M*^n^*^+^ → **[TC[4]A–M]^(4−n)−^**.

Complex	Δ*G*^1^	Δ*G*^78^	Δ*G*_exp_(water, pH 2, 25 °C)

**[C[4]A–Na]^3−^**	−265.5	−27.0	positive value [31]
**[C[4]A–Mg]^2−^**	−714.3	−59.4	−4.5 [30]
**[C[4]A–La]^−^**	−1102.3	45.3	−5.8 [30]
**[C[4]A–Rb]^3-^**	−215.5	18.9	−1.1 [31]
**[C[4]A–Sr]^2−^**	−599.9	19.7	
**[C[4]A–Lu]^−^**	−1183.1	−31.1	
			
**[TC[4]A–Na]^3−^**	−257.2	−25.8	
**[TC[4]A–Mg]^2−^**	−700.2	−57.7	
**[TC[4]A–La]^−^**	−1079.6	53.2	
**[TC[4]A–Rb]^3−^**	−200.8	24.9	
**[TC[4]A–Sr]^2−^**	−562.0	27.7	

**Table 2:** Metal cationic radii (Å).

*n*	metal cation M*^n^*^+^	ionic radius

1	Na^+^	0.99^a^/1.02^b^
	Rb^+^	1.52^b^
2	Mg^2+^	0.57^a^/0.72^b^
	Sr^2+^	1.18^b^
3	La^3+^	1.03 ^b^
	Lu^3+^	0.86 ^b^

^a^Ionic radius in tetracoordinated complexes; from Shannon, ref. [35]. ^b^Ionic radius in hexacoordinated complexes; from Shannon, ref. [35].

### **C[4]A** as a second-shell ligand: **[C[4]A–La(H_2_O)_9_]^−^** complex

Experimental (microcalorimetrical) studies on the complexation between *p*-sulfonatocalix[4]arene and different inorganic and organic cations (in water, at pH 2, 25 °C) revealed weak binding abilities for monovalent cations (it has been concluded that Na^+^ or Ag^+^ cations are not complexed by **C[4]A**) and moderate strong binding abilities for divalent and trivalent cations (Table 1) [30–31]. The discrepancies between the calculated Δ*G*^78^ values for **C[4]A** complex formation with the bare metal cations and the experimentally measured values provoked us to search for an explanation. A typical purely ionic (electrostatic) binding of **C[4]A** with metal cations has been suggested by Morel et al. and the important role of the desolvation of the species upon binding has been noted [31]. A model of hydrated, by an average number of 10 water molecules, La^3+^ cation has already been used by Mendes et al*.* in the MD simulations of *p*-sulfonatocalix[4]arene association with rare-earth metal cations and organic cations in aqueous solutions [32].

**Figure 6:**
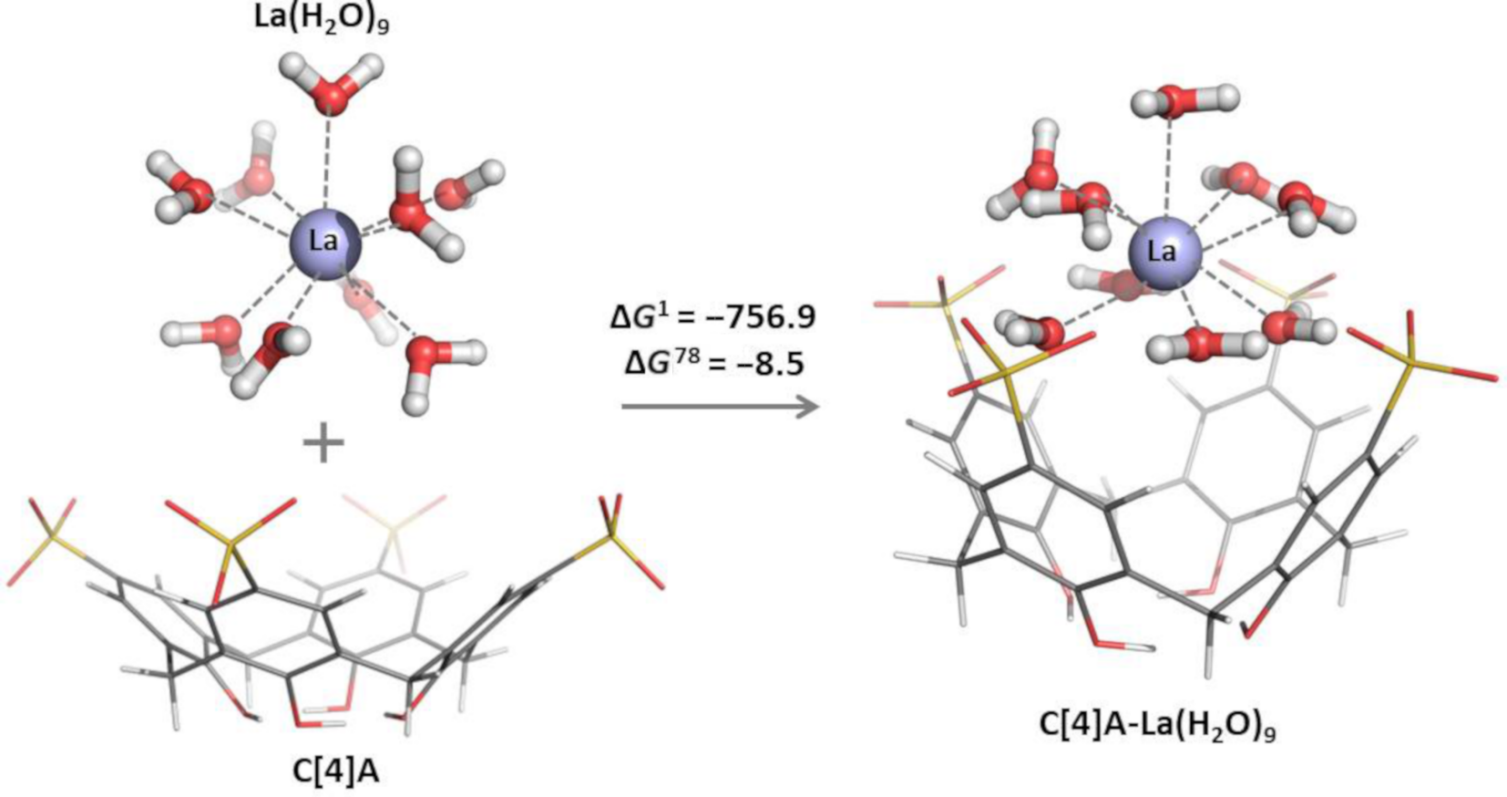
M062X/6-31G(d,p) optimized structures of the **[La(H_2_O)_9_]^3+^** cation, **C[4]A** host and **C[4]A** complex with hydrated metal cation. The BSSE-corrected Gibbs free energies Δ*G*^1^ and Δ*G*^78^ (in kcal/mol) for the complex formation reaction with hydrated metal cation are shown. Δ*G*^1^ refers to reaction free energy in the gas phase, whereas ΔG*^78^* refers to reaction free energies in an environment characterized by an effective dielectric constant of 78 (water).

The effect of metal hydration (i.e., explicit solvent treatment method) on the complexation process was studied here by employing a supramolecular approach for one representative of the metal species from the series, La^3+^ cation. A hydration number of 9 and initial tricapped trigonal prismatic arrangement of the water ligands were considered [36–37]. The optimized structure of the lanthanum nonaaqua complex, [La(H_2_O)_9_]^3+^ is shown in Figure 6.

The optimization of the **[La(H_2_O)_9_]^3+^** complex with **C[4]A** was initiated from the optimized geometries of both structures with **[La(H_2_O)_9_]^3+^** positioned above the cavity where the La^3+^ cation was approximately at the level of the SO_3_ groups. In the resultant complex (where **C[4]A** is a second-shell ligand) the cavity is not filled with water molecules, the four SO_3_ groups are attracted by the hydrated metal cation and the calixarene adopts a conformation with closer opposite cavity walls. The Gibbs free energies of complex formation in the gas phase (Δ*G*^1^) and water environment (Δ*G*^78^) are presented in Figure 6. The binding of **[La(H_2_O)_9_]^3+^** in the gas phase appears to be favorable, characterized with quite large negative Δ*G*^1^ value (−756.9 kcal mol^−1^). The negative Δ*G*^78^ value (−8.5 kcal mol^−1^) implies that the complex formation reaction in aqueous environment with incoming hydrated metal cation is also favorable. This value is in good agreement with the experimentally derived one by Bonal et al. in water at 298.15 K and at pH 2 (−24.1 kJ mol^−1^ or −5.8 kcal mol^−1^) [30].

## Conclusion

A systematic theoretical study of the group IA, IIA and f-block metal ions binding characteristics to *p*-sulfonatocalix[4]arene (**C[4]A**) and *p*-sulfonatothiacalix[4]arene (**TC[4]A**) has been performed using density functional theory combined with solvation model based on density (SMD) calculations. It is shown that the metal cations induce different pre-organization of the calixarene structure upon binding. The preferred binding site for the guest metal cations is the plane of the upper rim (with *p*-sulfonato groups), i.e., the sulfonate groups of hosts serve as anchoring points for the positively charged guests. The negative values calculated for the Gibbs energies of the **C[4]A** and **TC[4]A** – IA group/IIA group/f-block metal cations complexation process are indicative for a spontaneous and exergonic (energy-favorable) process in the gas phase for all metal cations and for three of the cations (Na^+^, Mg^2+^, Lu^3+^) in aqueous environment. The **C[4]A** seems to possess a slightly higher metal affinity than its **TC[4]A** counterpart (Table 1) although the overall behavior of the two host calixarenes toward metal guests are similar. The implicit solvent treatment alone is not enough to represent the state of the system in solution, in particular in a very polar medium like water. The combination of implicit/explicit solvent treatment provides a more realistic description of the behavior of the *p-*sulfonatocalix[4]arene host system and metal cations in water solution and makes the evaluation of the thermodynamic parameters of the complex formation reaction meaningful.

### Computational

The molecules of the ligands (calix[4]arenes and thiacalix[4]arenes), group IA, IIA and f-block metal cations and their complexes were optimized using the Gaussian 09 program package [38]. The computations were performed with the 6-31G(d,p) [39–40] basis set for the lighter atoms (C, O, S, H, Na, and Mg) and SDD [41–42] pseudopotential for Rb, Sr, La and Lu. The M062X/6-31G(d,p);SDD combination method/basis set was chosen because it performed well in reproducing the experimental structural characteristics of appropriate model entities: The experimental metal–oxygen bond distance (Na–O_SO3_) in the poly[μ2-aqua-(μ3-2,5-dichlorobenzenesulfonato)sodium, [Na(C_6_H_3_Cl_2_O_3_S)(H_2_O)]*_n_* [43] (2.2974 Å), was reliably reproduced at the M062X/6-31G(d,p) level where the calculated Na^+^–O_SO3_ distance in the modeled [Na(C_6_H_3_Cl_2_O_3_S)(H_2_O)_4_] complex is 2.2663 Å (present work, Supporting Information File 1). Frequency calculations for each optimized structure were performed at the same level of theory. The full set of positive frequencies obtained for each metal complex indicated a local minimum on the potential energy surface.

The differences between the products and reactants of electronic energies, Δ*E*_el_, thermal energies, incorporating zero-point energy, Δ*E*_th_, and entropies, Δ*S*, in the gas phase (ε = 1) were used to evaluate the Gibbs energy of the complex formation, ΔG^1^, at *T* = 298.15 K according to the equation:

[1] $$\Delta G^{1}=\Delta E_{\text{el}}+\Delta E_{\text{th}}+P\Delta V–T\Delta S$$

The counterpoise procedure of Boys and Bernardi [44] was utilized to correct for basis set superposition errors (BSSE) as coded in Gaussian 09 package.

Solvation effects were evaluated by employing the solvation model based on density (SMD) [45] as implemented in the Gaussian 09 suite of programs. The fully optimized structure of the respective reactant or product of the reaction in the gas phase was subjected to a single point calculation in water (with dielectric constant ε = 78). The difference between the gas-phase and SMD energies yielded the solvation energy, ∆*E*_solv_^ε^ ≈ ∆*G*_solv_^ε^, of the molecule/complex. Solvation free energies of the products and reactants were used to calculate the free energy of the complex formation in condensed medium (water):

[2]
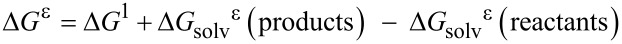


The fully optimized structure of some molecules and complexes in the gas phase was also re-optimized in water (with a dielectric constant ε = 78). The ∆*G* values derived from Equation 1 using the respective Δ*E*_el_, Δ*E*_th_ and Δ*S* values for the optimized in water structures are presented in Table S2, Supporting Information File 1.

A thermodynamically unfavorable complex formation (in the gas phase or in condensed medium) is indicated by a positive ∆*G*^ε^ value, whereas a favorable one is indicated by a negative ∆*G*^ε^ value.

PyMOL molecular graphics system was used for generation of the molecular graphics images [46].

## Supporting Information

File 1 Validation of the chosen computational level, additional structural data and thermodynamic parameters for the complex formation reactions.
