# Supplementary material for: Host–guest interactions between p-sulfonatocalix[4]arene and p-sulfonatothiacalix[4]arene and group IA, IIA and f-block metal cations: a DFT/SMD study
Source: Beilstein J Org Chem. 2019 Jun 17;15:1321–30. doi: 10.3762/bjoc.15.131 (PMC6604682; doi:10.3762/bjoc.15.131)
Supplement: File 1 — Validation of the chosen computational level, additional structural data and thermodynamic parameters for the complex formation reactions. [file Beilstein_J_Org_Chem-15-1321-s001.pdf]

BEILSTEIN JOURNAL OF ORGANIC CHEMISTRY

Supporting Information

for

Host–guest interactions between *p*-sulfonatocalix[4]arene and *p*-sulfonatothiacalix[4]arene and group IA, IIA and f-block metal cations: a DFT/SMD study

Valya K. Nikolova, Cristina V. Kirkova, Silvia E. Angelova and Todor M. Dudev

Beilstein J. Org. Chem. **2019,** *15,* 1321–1330. doi:10.3762/bjoc.15.131

Validation of the chosen computational level, additional structural data and thermodynamic parameters for the complex formation reactions

License and Terms: This is a supporting information file under the terms of the Creative Commons Attribution License (http://creativecommons.org/licenses/by/4.0). Please note that the reuse, redistribution and reproduction in particular requires that the authors and source are credited.
The license is subject to the *Beilstein Journal of Organic Chemistry* terms and conditions: (https://www.beilstein-journals.org/bjoc)

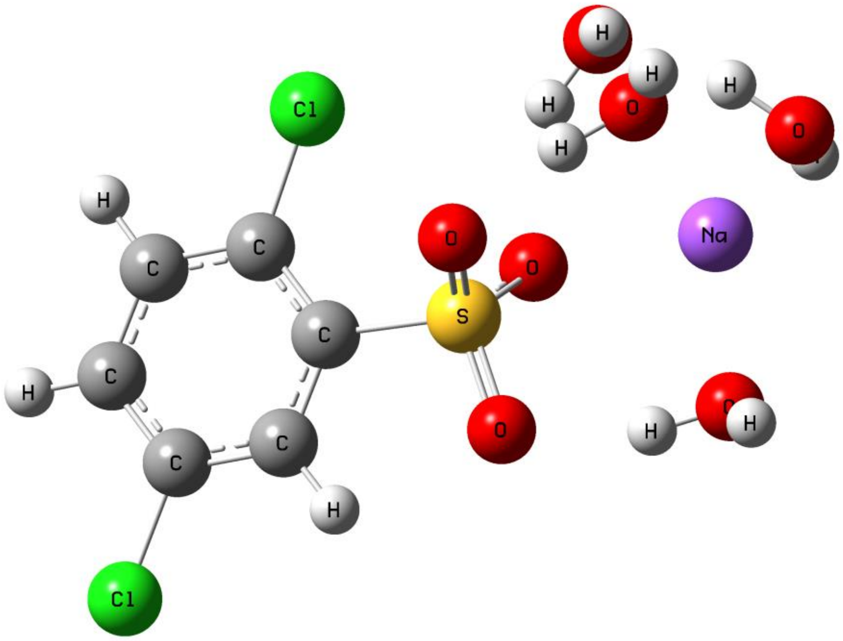

Figure S1: Optimized structure of [Na(H_2O)$_4$(sulfonato-2,5-dichlorobenzene] complex.

Table S1: Experimental Na–O(SO_3) distance in poly[μ2-aqua-(μ3-2,5-dichlorobenzenesulfonato)sodium], [Na($C_6H_3Cl_2O_3S$)(H_2O)]$_n$, and the calculated one in the optimized at different computational levels [Na($C_6H_3Cl_2O_3S$)(H_2O)$_4$] complex.

	Na-O(SO_3) distance, Å	Exp. – calc., Å
Exp.	2.2974	
HF/6-31G(d,p)	2.3084	-0.0110
HF/6-31+G(d,p)	2.3168	-0.0194
B3LYP/6-31G(d,p)	2.3344	-0.0370
B3LYP /6-31+G(d,p)	2.3320	-0.0346
**M062X/6-31G(d,p)**	**2.2663**	**0.0311**
M062X /6-31+G(d,p)	2.2658	0.0316

Table S2: Calculated Gibbs free energies (in kcal mol^{-1}) in the gas phase (ΔG^{1}) and in water (ΔG^{78}; single point calculations and optimization) for the **[C[4]A−M]$^{n+}$** and **[TC[4]A−M]$^{n+}$** (n = 1–3) complex formation reactions, **C[4]A + M^{n+} → [C[4]A−M]$^{n+}$** and **TC[4]A** + M^{n+} → **[TC[4]A−M]$^{n+}$**.

	Gas phase		SP calculations (water)		Full optimization (water)	
Complex	ΔG^{1}	ΔG^{1} (BSSE corrected)	ΔG^{78}	ΔG^{78} (BSSE corrected)	ΔG^{78}	ΔG^{78} (BSSE corrected)
**C[4]A-Na**¬$^{+}$	-272.6	-265.5	-34.1	-27.0	-31.7	-24.6
**C[4]A-Mg**¬$^{2+}$	-723.1	-714.3	-68.1	-59.4	-64.6	-55.9
**C[4]A-La**¬$^{3+}$	-1114.0	-1102.3	33.6	45.3	-	-
**C[4]A-Rb**¬$^{+}$	-220.8	-215.5	13.6	18.9	15.9	21.1
**C[4]A-Sr**¬$^{2+}$	-611.8	-599.9	7.7	19.7	6.9	18.8
**C[4]A-Lu**¬$^{3+}$	-1194.8	-1183.1	-42.8	-31.1	-	-
**TC[4]A-Na**¬$^{+}$	-264.1	-257.2	-32.8	-25.8	-	-
**TC[4]A-Mg**¬$^{2+}$	-708.9	-700.2	-66.4	-57.7	-	-
**TC[4]A-La**¬$^{3+}$	-1091.1	-1079.6	41.7	53.2		
**TC[4]A-Rb**¬$^{+}$	-206.0	-200.8	19.8	24.9	-	-
**TC[4]A-Sr**¬$^{2+}$	-573.4	-562.0	16.3	27.7	-	-
**TC[4]A-Lu**¬$^{3+}$	-1172.8	-1161.2	-36.2	-24.6	-	-

Table S3: Thermodynamic parameters (in kcal mol^{-1}) in the gas phase calculated for the **[C[4]A−M]$^{n+}$** and **[TC[4]A−M]$^{n+}$** (n = 1–3) complex formation reactions, **C[4]A + M^{n+}** → **[C[4]A−M]$^{n+}$** and **TC[4]A** + M^{n+} → **[TC[4]A−M]$^{n+}$**.

Complex	ΔE_{el}^{1}	ΔH^{1}	$T\Delta S^{1}$	ΔG^{1}
**C[4]A-Na┐$^{+}$**	-281.9	-280.1	-7.5	-272.6
**C[4]A-Mg┐$^{2+}$**	-736.9	-734.2	-11.2	-723.1
**C[4]A-La┐$^{3+}$**	-1129.9	-1127.2	-13.2	-1114.0
**C[4]A-Rb┐$^{+}$**	-230.4	-228.5	-7.7	-220.8
**C[4]A-Sr┐$^{2+}$**	-625.7	-623.1	-11.3	-611.8
**C[4]A-Lu┐$^{3+}$**	-1211.4	-1208.3	-13.5	-1194.8
**TC[4]A-Na┐$^{+}$**	-274.9	-273.2	-9.1	-264.1
**TC[4]A-Mg┐$^{2+}$**	-723.8	-721.5	-12.6	-708.9
**TC[4]A-La┐$^{3+}$**	-1105.8	-1104.3	-13.2	-1091.1
**TC[4]A-Rb┐$^{+}$**	-216.1	-214.4	-8.5	-206.0
**TC[4]A-Sr┐$^{2+}$**	-585.9	-584.0	-10.6	-573.4
**TC[4]A-Lu┐$^{3+}$**	-1188.7	-1187.2	-16.2	-1171.0

Table S4: Cartesian coordinates for the gas phase optimized structure of **C[4]A**".

	0.764678000	1.613579000	2.581214000
O	1.613829000	-0.765482000	2.581930000
O	-0.765377000	-1.613749000	2.581712000
C	2.103865000	4.180267000	-0.359499000
C	2.924772000	3.138994000	0.062381000
C	2.481369000	2.200783000	0.997690000
C	1.181335000	2.358181000	1.498972000
C	3.428370000	1.133308000	1.545236000
C	3.317466000	-0.289570000	1.001836000
C	4.235156000	-0.781261000	0.072551000
C	4.180507000	-2.103407000	-0.359235000
C	3.139375000	-2.924587000	0.062456000
C	2.200929000	-2.481531000	0.997690000
C	2.358214000	-1.181657000	1.499366000
C	1.133410000	-3.428764000	1.544802000
C	-0.289577000	-3.317549000	1.001570000
C	-0.781484000	-4.235113000	0.072311000
C	-2.103596000	-4.180201000	-0.359586000
C	-1.181534000	-2.358203000	1.499165000
H	3.938805000	3.089221000	-0.326688000
H	3.319047000	1.097866000	2.637952000
H	4.447544000	1.476911000	1.345933000
H	5.027831000	-0.143527000	-0.310805000
H	3.089827000	-3.938535000	-0.326863000
H	1.098218000	-3.320470000	2.637612000
H	1.476916000	-4.447775000	1.344491000

H	-0.143845000	-5.027842000	-0.311078000
O	-1.613264000	0.765333000	2.581128000
C	0.781692000	4.235246000	0.072172000
C	0.289565000	3.317621000	1.001263000
C	-1.133477000	3.428862000	1.544320000
C	-2.200936000	2.481547000	0.997249000
C	-3.139579000	2.924645000	0.062228000
C	-4.180758000	2.103477000	-0.359336000
C	-4.235327000	0.781316000	0.072420000
C	-3.317470000	0.289580000	1.001512000
C	-2.358037000	1.181614000	1.498847000
C	-3.428453000	-1.133278000	1.544971000
C	-2.481408000	-2.200772000	0.997571000
C	-2.924620000	-3.138972000	0.062153000
H	0.144204000	5.028062000	-0.311282000
H	-0.721360000	1.226203000	2.601342000
H	-1.098377000	3.320689000	2.637144000
H	-1.476989000	4.447846000	1.343873000
H	-3.090169000	3.938638000	-0.326990000
H	-5.028084000	0.143602000	-0.310800000
H	-3.319200000	-1.097767000	2.637693000
H	-4.447618000	-1.476881000	1.345621000
H	-3.938596000	-3.089286000	-0.327071000
H	1.225287000	0.721520000	2.601447000
H	0.721952000	-1.226430000	2.602418000
H	-1.226277000	-0.721852000	2.601996000
S	-5.569087000	2.808164000	-1.281670000
O	-6.482679000	3.292521000	-0.221270000
O	-4.980564000	3.894340000	-2.089692000
S	-2.808391000	-5.568031000	-1.282590000
O	-1.682015000	-6.118410000	-2.062312000
O	-3.293202000	-6.481957000	-0.222700000
S	5.568620000	-2.808104000	-1.281887000
O	6.119586000	-1.681584000	-2.060999000
O	6.481952000	-3.293434000	-0.221710000
S	2.808932000	5.567966000	-1.282487000
O	3.295542000	6.480969000	-0.222626000
O	3.893477000	4.978378000	-2.091911000
O	-6.119573000	1.681899000	-2.061482000
O	-3.894220000	-4.978925000	-2.090647000
O	1.682231000	6.119649000	-2.060830000
O	4.979736000	-3.893577000	-2.090587000

Table S5: Cartesian coordinates for the gas phase optimized structure of TC[4]A.

O	-1.830593000	0.032508000	-2.305458000
O	-0.014726000	-1.833512000	-2.313328000
O	1.852684000	-0.014019000	-2.300558000
C	-4.952995000	-0.066143000	0.416963000
C	-4.365426000	-1.257405000	0.003495000
C	-3.270938000	-1.255674000	-0.864646000
C	-2.775376000	-0.026004000	-1.336205000
C	-1.184121000	-3.308558000	-0.874469000
C	-1.159593000	-4.401395000	-0.005441000
C	0.046989000	-4.944480000	0.423564000
C	1.243586000	-4.358602000	0.023739000
C	1.253334000	-3.268022000	-0.849540000
C	0.030240000	-2.774511000	-1.339248000
C	3.314611000	-1.184788000	-0.848380000
C	4.396397000	-1.161344000	0.033503000
C	4.938336000	0.045502000	0.464181000
C	2.787977000	0.030760000	-1.320865000
H	-4.806889000	-2.199838000	0.312603000
H	-2.086569000	-4.874486000	0.302760000
H	2.181555000	-4.798912000	0.347622000
H	4.838893000	-2.089766000	0.381331000
O	0.031391000	1.855732000	-2.298924000
C	-4.407743000	1.145999000	0.007065000
C	-3.311382000	1.181829000	-0.857428000
C	-1.255415000	3.271657000	-0.831893000
C	-1.256343000	4.349074000	0.058177000
C	-0.065154000	4.933907000	0.472849000
C	1.147423000	4.392793000	0.057513000
C	1.182329000	3.313370000	-0.827241000
C	-0.026692000	2.788281000	-1.317468000
C	3.272404000	1.252762000	-0.820101000
C	4.352160000	1.242056000	0.067372000
H	-4.881805000	2.068708000	0.326373000
H	-0.812822000	1.343620000	-2.362189000
H	-2.195674000	4.758414000	0.417207000
H	2.071124000	4.834216000	0.418947000
H	4.762643000	2.176732000	0.437112000
H	-1.318931000	-0.812317000	-2.367424000
H	0.831357000	-1.323282000	-2.366315000
H	1.343983000	0.832618000	-2.357092000
S	-2.812330000	2.755288000	-1.571543000
S	-2.748363000	-2.808728000	-1.608231000
S	2.815576000	-2.751244000	-1.577551000
S	2.757599000	2.819069000	-1.540378000
S	-6.474330000	-0.098642000	1.405015000
O	-7.114000000	1.196485000	1.093998000
O	-7.196914000	-1.273637000	0.874865000

S	-0.094292000	6.494230000	1.393580000
O	1.152761000	6.481057000	2.181465000
O	-0.117594000	7.524576000	0.333789000
S	6.500383000	0.060109000	1.382483000
O	6.489061000	-1.199661000	2.149587000
O	7.528777000	0.099798000	0.321329000
S	0.064133000	-6.460490000	1.420291000
O	-1.214935000	-7.113705000	1.072505000
O	1.261835000	-7.174374000	0.930800000
O	-6.047651000	-0.240523000	2.807457000
O	-1.336131000	6.431299000	2.186637000
O	0.158116000	-6.025043000	2.824000000
O	6.439353000	1.288577000	2.196520000
